# 2,2′-[1,2-Phenyl­enebis(aza­nedi­yl)]di­aceto­nitrile

**DOI:** 10.1107/S1600536812047538

**Published:** 2012-11-24

**Authors:** Augusto Rivera, Leonardo Jiménez-Cruz, Mauricio Maldonado, Monika Kučeráková, Michal Dušek

**Affiliations:** a Universidad Nacional de Colombia, Sede Bogotá, Facultad de Ciencias, Departamento de Química, Cra 30 No. 45-03, Bogotá, Código Postal 111321, Colombia; b Institute of Physics ASCR, v.v.i., Na Slovance 2, 182 21 Praha 8, Czech Republic

## Abstract

The title compound, C_10_H_10_N_4_, shows chemical but not crystallographic *C*_2_ symmetry. The two cyano­methyl groups are located in an *anti* position with respect to the aromatic ring. In the crystal, mol­ecules form parallel ladder-like tapes linked through two N—H⋯N hydrogen bonds. Further weak intra­molecular N—H⋯N hydrogen bonding is responsible for the elongation of one of the C_aromatic_—N bonds.

## Related literature

For general background to the title compound, see: Rivera *et al.* (2010 ▶). For related structures, see: Rivera *et al.* (2010 ▶, 2011 ▶); Silversides *et al.* (2006 ▶).
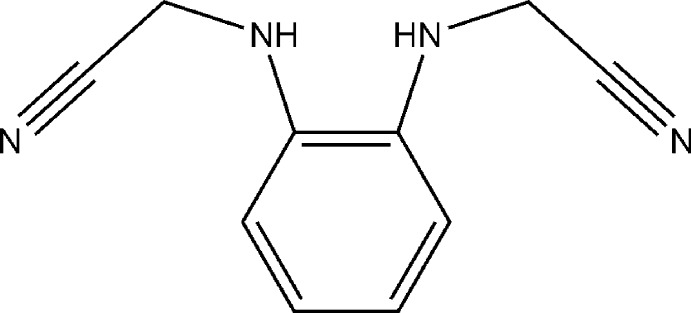


## Experimental

### 

#### Crystal data

C_10_H_10_N_4_
*M* *_r_* = 186.2
Orthorhombic, 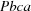

*a* = 7.6404 (3) Å
*b* = 15.1703 (7) Å
*c* = 15.9168 (7) Å
*V* = 1844.87 (14) Å^3^
*Z* = 8
Cu *K*α radiation
μ = 0.69 mm^−1^
*T* = 120 K
0.17 × 0.15 × 0.10 mm

#### Data collection

Agilent Xcalibur (Atlas, Gemini ultra) diffractometer
Absorption correction: multi-scan (*CrysAlis PRO*; Agilent, 2012 ▶) *T*_min_ = 0.68, *T*_max_ = 1
9893 measured reflections
1641 independent reflections
1359 reflections with *I* > 3σ(*I*)
*R*_int_ = 0.061

#### Refinement

*R*[*F* ^2^ > 2σ(*F* ^2^)] = 0.039
*wR*(*F* ^2^) = 0.104
*S* = 1.55
1641 reflections
133 parameters
H atoms treated by a mixture of independent and constrained refinement
Δρ_max_ = 0.16 e Å^−3^
Δρ_min_ = −0.20 e Å^−3^

### 

Data collection: *CrysAlis PRO* (Agilent, 2012 ▶); cell refinement: *CrysAlis PRO*; data reduction: *CrysAlis PRO*; program(s) used to solve structure: *SUPERFLIP* (Palatinus & Chapuis, 2007 ▶); program(s) used to refine structure: *JANA2006* (Petříček *et al.*, 2006 ▶); molecular graphics: *DIAMOND* (Brandenburg & Putz, 2005 ▶); software used to prepare material for publication: *JANA2006*.

## Supplementary Material

Crystal structure: contains datablock(s) global, I. DOI: 10.1107/S1600536812047538/bt6866sup1.cif

Structure factors: contains datablock(s) I. DOI: 10.1107/S1600536812047538/bt6866Isup2.hkl

Supplementary material file. DOI: 10.1107/S1600536812047538/bt6866Isup3.cml

Additional supplementary materials:  crystallographic information; 3D view; checkCIF report

## Figures and Tables

**Table 1:** Hydrogen-bond geometry (Å, °)

*D*—H⋯*A*	*D*—H	H⋯*A*	*D*⋯*A*	*D*—H⋯*A*
N1—H1⋯N4^i^	0.935 (18)	2.202 (19)	3.0946 (19)	159.2 (16)
N2—H2⋯N1	0.889 (18)	2.427 (18)	2.7524 (18)	102.0 (13)
N2—H2⋯N1^ii^	0.889 (18)	2.494 (17)	3.2536 (16)	143.8 (15)

Symmetry codes: (i) 
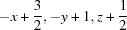
; (ii) 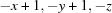.

**Fig. 1.**
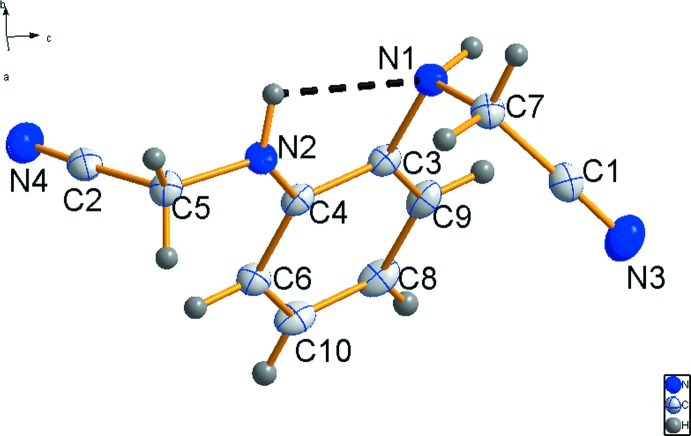
A view of the crystal structure of the tittle compund (I) with the numbering scheme. Displacement ellipsoids are drawn at the 50% probability level.

**Fig. 2.**
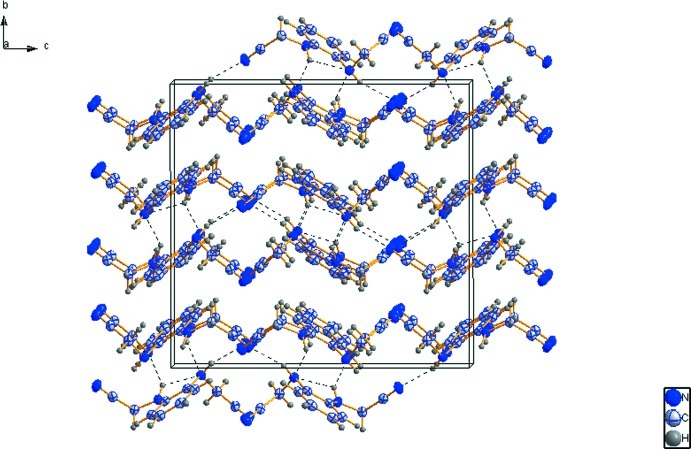
Packing of the molecules of the title compound view along a axis. N—H···CN and N—H···N hydrogen bonds are drawn as dashed lines.

## supplementary crystallographic information

### Comment

The reaction of *N*^1^,*N*^2^-bis((1*H*-benzotriazol-1-yl)methyl)benzene-1,2-diamine with potassium cyanide in DMSO at room temperature gives the title compound in high yield. The two cyanomethyl groups are both *anti* with respect to the planar aromatic ring, no doubt in order to favour intermolecular N—H···N interactions. Intermolecular amine-to-amine and amine-to-cyano hydrogen bonding interactions established "ladder-like" parallel tapes in the crystalline solid (Fig. 2).

The molecular structure and atom numbering scheme for the title compound are shown in Fig. 1. Its X-ray structure confirms the presence of intramolecular hydrogen bond between the amino groups [N–H, 2.426 (18) Å]. Furthermore the observed C3—N1 bond length [1.4357 (17) Å] is considerably elongated in relation to related structures [1.404 (3) Å] (Rivera *et al.*, 2010), [1.3961 (18) Å] (Silversides *et al.*, 2006) and is longer than the C4—N2 bond [1.4016 (16) Å]. Thus, these results indicate that the intramolecular interaction is responsible for the C3—N1 bond elongation. This is confirmed by the N1—C7 bond length [1.4648 (18) Å] that is longer than N2—C5 bond [1.4457 (19) Å]. Besides, the hybridization of both N1 and N2 atoms are slightly different. In fact, N1 has more *sp^2^* character [Sα = 347.25°] than N2 [Sα = 330.39°] if compared to *sp^3^*-hybridization [Sα = 328.50°] angle value. Other bond distances and bond angles are in good agreement with the standard values.

### Experimental

A mixture of potassium cyanide (0.260 g, 4.00 mmol) and *N*^1^,*N*^2^-bis((1*H*-benzotriazol-1-yl)methyl)benzene-1,2-diamine (0.370 g, 1.00 mmol) was stirred at room temperature in DMSO (5 ml) for 6 h. After quenching by addition of aqueous ammonium chloride a solid formed which was separated and extracted with ethyl acetate.The organic extract was sequentially washed with saturated Na_2_CO_3_ solution and water, then dried (Na_2_SO_4_), filtered, and concentrated to give a solid homogeneous by TLC (80% EtOAc/benzene) in 82% yield. Single crystals were obtained by recrystallization from chloroform/methanol by slow evaporation of the solvent at room temperaature, m.p. 399–400 K.

### Refinement

All hydrogen atoms were discernible in difference Fourier maps and could be refined to reasonable geometry, but according to common practice H atoms bonded to C were kept in ideal positions with C–H = 0.96 Å. The coordinates of the amino H atoms were refined. *U*_iso_(H) was set to 1.2*U*_eq_(C,N). All non-hydrogen atoms were refined using harmonic refinement.

### Crystal data

**Table:**

C_10_H_10_N_4_	*D*_x_ = 1.341 Mg m^−^^3^
*M**_r_* = 186.2	Melting point: 399 K
Orthorhombic, *P**b**c**a*	Cu *K*α radiation, λ = 1.5418 Å
Hall symbol: -P 2ac 2ab	Cell parameters from 4237 reflections
*a* = 7.6404 (3) Å	θ = 4.0–67.0°
*b* = 15.1703 (7) Å	µ = 0.69 mm^−^^1^
*c* = 15.9168 (7) Å	*T* = 120 K
*V* = 1844.87 (14) Å^3^	Polygon shape, white
*Z* = 8	0.17 × 0.15 × 0.10 mm
*F*(000) = 784	

### Data collection

**Table:**

Agilent Xcalibur (Atlas, Gemini ultra) diffractometer	1641 independent reflections
Radiation source: Enhance Ultra (Cu) X-ray Source	1359 reflections with *I* > 3σ(*I*)
Mirror monochromator	*R*_int_ = 0.061
Detector resolution: 10.3784 pixels mm^-1^	θ_max_ = 67.2°, θ_min_ = 5.6°
ω scans	*h* = −7→9
Absorption correction: multi-scan (*CrysAlis PRO*; Agilent, 2012)	*k* = −18→18
*T*_min_ = 0.68, *T*_max_ = 1	*l* = −18→16
9893 measured reflections	

### Refinement

**Table:**

Refinement on *F*^2^	34 constraints
*R*[*F*^2^ > 2σ(*F*^2^)] = 0.039	H atoms treated by a mixture of independent and constrained refinement
*wR*(*F*^2^) = 0.104	Weighting scheme based on measured s.u.'s *w* = 1/(σ^2^(*I*) + 0.0016*I*^2^)
*S* = 1.55	(Δ/σ)_max_ = 0.005
1641 reflections	Δρ_max_ = 0.16 e Å^−^^3^
133 parameters	Δρ_min_ = −0.20 e Å^−^^3^
0 restraints	

### Special details

**Table:**

Refinement. The refinement was carried out against all reflections. The conventional *R*-factor is always based on *F*. The goodness of fit as well as the weighted *R*-factor are based on *F* and *F*^2^ for refinement carried out on *F* and *F*^2^, respectively. The threshold expression is used only for calculating *R*-factors *etc*. and it is not relevant to the choice of reflections for refinement.The program used for refinement, Jana2006, uses the weighting scheme based on the experimental expectations, see _refine_ls_weighting_details, that does not force *S* to be one. Therefore the values of *S* are usually larger than the ones from the *SHELX* program.

### Fractional atomic coordinates and isotropic or equivalent isotropic displacement parameters (Å^2^)

**Table:**

	*x*	*y*	*z*	*U*_iso_*/*U*_eq_	
N1	0.67776 (14)	0.46314 (7)	0.09105 (8)	0.0197 (3)	
N2	0.59009 (13)	0.37473 (8)	−0.05397 (8)	0.0182 (3)	
N3	0.75959 (16)	0.31452 (9)	0.25065 (9)	0.0316 (4)	
N4	0.68740 (17)	0.42625 (9)	−0.25896 (9)	0.0314 (4)	
C1	0.67383 (17)	0.35295 (9)	0.20428 (10)	0.0228 (4)	
C2	0.62292 (17)	0.38628 (9)	−0.20642 (10)	0.0227 (4)	
C3	0.81190 (16)	0.41747 (8)	0.04529 (9)	0.0179 (4)	
C4	0.76583 (16)	0.37282 (8)	−0.02878 (9)	0.0176 (4)	
C5	0.54231 (16)	0.33892 (9)	−0.13496 (9)	0.0200 (4)	
C6	0.89448 (16)	0.32671 (9)	−0.07229 (10)	0.0203 (4)	
C7	0.57084 (17)	0.40361 (9)	0.14220 (10)	0.0215 (4)	
C8	1.11225 (17)	0.37219 (9)	0.02841 (11)	0.0248 (4)	
C9	0.98466 (17)	0.41713 (9)	0.07282 (10)	0.0224 (4)	
C10	1.06689 (17)	0.32645 (10)	−0.04320 (10)	0.0238 (4)	
H1c5	0.417265	0.339719	−0.140823	0.024*	
H2c5	0.57431	0.277778	−0.137292	0.024*	
H1c6	0.864639	0.294979	−0.122465	0.0244*	
H1c7	0.508788	0.363609	0.106188	0.0258*	
H2c7	0.481985	0.436862	0.17073	0.0258*	
H1c8	1.23154	0.372856	0.047341	0.0298*	
H1c9	1.015681	0.44837	0.123117	0.0268*	
H1c10	1.154716	0.294105	−0.073369	0.0286*	
H1	0.732 (2)	0.5050 (12)	0.1255 (12)	0.0237*	
H2	0.536 (2)	0.4245 (12)	−0.0403 (12)	0.0219*	

### Atomic displacement parameters (Å^2^)

**Table:**

	*U*^11^	*U*^22^	*U*^33^	*U*^12^	*U*^13^	*U*^23^
N1	0.0140 (5)	0.0231 (6)	0.0221 (7)	0.0001 (4)	−0.0013 (5)	−0.0013 (5)
N2	0.0087 (5)	0.0257 (6)	0.0202 (7)	0.0023 (4)	0.0000 (5)	−0.0013 (5)
N3	0.0229 (6)	0.0399 (7)	0.0319 (8)	−0.0047 (6)	−0.0020 (6)	0.0091 (6)
N4	0.0267 (6)	0.0389 (7)	0.0287 (8)	0.0053 (6)	0.0054 (6)	0.0065 (6)
C1	0.0167 (7)	0.0287 (7)	0.0231 (8)	−0.0035 (6)	0.0041 (6)	−0.0004 (6)
C2	0.0154 (6)	0.0286 (7)	0.0241 (9)	0.0052 (6)	−0.0007 (6)	−0.0013 (6)
C3	0.0117 (6)	0.0210 (6)	0.0210 (8)	−0.0002 (5)	0.0014 (5)	0.0041 (5)
C4	0.0101 (6)	0.0217 (6)	0.0211 (8)	−0.0006 (5)	0.0001 (5)	0.0045 (5)
C5	0.0124 (6)	0.0268 (7)	0.0208 (8)	−0.0011 (5)	−0.0006 (6)	0.0008 (5)
C6	0.0131 (6)	0.0249 (7)	0.0229 (8)	0.0011 (5)	0.0019 (6)	0.0012 (6)
C7	0.0137 (6)	0.0285 (7)	0.0223 (8)	0.0013 (5)	0.0007 (6)	−0.0007 (6)
C8	0.0094 (6)	0.0300 (7)	0.0350 (9)	−0.0019 (5)	−0.0030 (6)	0.0081 (6)
C9	0.0153 (6)	0.0262 (7)	0.0256 (9)	−0.0038 (5)	−0.0042 (6)	0.0051 (6)
C10	0.0107 (6)	0.0284 (7)	0.0324 (9)	0.0028 (5)	0.0043 (6)	0.0064 (6)

### Geometric parameters (Å, º)

**Table:**

N1—C3	1.4357 (17)	C4—C6	1.3911 (19)
N1—C7	1.4648 (18)	C5—H1c5	0.96
N1—H1	0.935 (18)	C5—H2c5	0.96
N2—C4	1.4016 (16)	C6—C10	1.3963 (18)
N2—C5	1.4457 (19)	C6—H1c6	0.96
N2—H2	0.889 (18)	C7—H1c7	0.96
N3—C1	1.146 (2)	C7—H2c7	0.96
N4—C2	1.144 (2)	C8—C9	1.384 (2)
C1—C7	1.479 (2)	C8—C10	1.379 (2)
C2—C5	1.480 (2)	C8—H1c8	0.96
C3—C4	1.404 (2)	C9—H1c9	0.96
C3—C9	1.3908 (18)	C10—H1c10	0.96
			
C3—N1—C7	112.49 (10)	H1c5—C5—H2c5	105.18
C3—N1—H1	108.1 (10)	C4—C6—C10	120.20 (14)
C7—N1—H1	109.8 (11)	C4—C6—H1c6	119.9
C4—N2—C5	119.29 (11)	C10—C6—H1c6	119.9
C4—N2—H2	113.2 (11)	N1—C7—C1	113.27 (11)
C5—N2—H2	114.8 (12)	N1—C7—H1c7	109.47
N3—C1—C7	177.27 (15)	N1—C7—H2c7	109.47
N4—C2—C5	176.55 (16)	C1—C7—H1c7	109.47
N1—C3—C4	118.67 (11)	C1—C7—H2c7	109.47
N1—C3—C9	121.29 (13)	H1c7—C7—H2c7	105.38
C4—C3—C9	120.04 (12)	C9—C8—C10	119.55 (13)
N2—C4—C3	118.05 (12)	C9—C8—H1c8	120.22
N2—C4—C6	123.02 (13)	C10—C8—H1c8	120.22
C3—C4—C6	118.90 (12)	C3—C9—C8	120.63 (14)
N2—C5—C2	113.45 (11)	C3—C9—H1c9	119.69
N2—C5—H1c5	109.47	C8—C9—H1c9	119.69
N2—C5—H2c5	109.47	C6—C10—C8	120.65 (13)
C2—C5—H1c5	109.47	C6—C10—H1c10	119.67
C2—C5—H2c5	109.47	C8—C10—H1c10	119.67

### Hydrogen-bond geometry (Å, º)

**Table:**

*D*—H···*A*	*D*—H	H···*A*	*D*···*A*	*D*—H···*A*
N1—H1···N4^i^	0.935 (18)	2.202 (19)	3.0946 (19)	159.2 (16)
N2—H2···N1	0.889 (18)	2.427 (18)	2.7524 (18)	102.0 (13)
N2—H2···N1^ii^	0.889 (18)	2.494 (17)	3.2536 (16)	143.8 (15)

Symmetry codes: (i) −*x*+3/2, −*y*+1, *z*+1/2; (ii) −*x*+1, −*y*+1, −*z*.
